# Complete resection versus functional preservation in resection of cystic vestibular schwannoma in a 56-year-old female: case report and literature review

**DOI:** 10.1093/jscr/rjad655

**Published:** 2023-12-14

**Authors:** Nyoman Golden, Steven Awyono, Dicky T Prakoso, Christopher Lauren

**Affiliations:** Neurosurgery Division, Department of Surgery, Faculty of Medicine, Universitas Udayana, Prof. Dr. I.G.N.G. Ngoerah General Hospital, Denpasar, Bali, Indonesia; Neurosurgery Division, Department of Surgery, Faculty of Medicine, Universitas Udayana, Prof. Dr. I.G.N.G. Ngoerah General Hospital, Denpasar, Bali, Indonesia; Neurosurgery Division, Department of Surgery, Faculty of Medicine, Universitas Udayana, Prof. Dr. I.G.N.G. Ngoerah General Hospital, Denpasar, Bali, Indonesia; Neurosurgery Division, Department of Surgery, Faculty of Medicine, Universitas Udayana, Prof. Dr. I.G.N.G. Ngoerah General Hospital, Denpasar, Bali, Indonesia

**Keywords:** functional preservation, internal auditory meatus, neurosurgery, oncology, vestibular schwannoma

## Abstract

Surgery for vestibular schwannoma presents unique challenges to the surgeon, given that the primary objectives are achieving complete resection while preserving both facial nerve and hearing function. Consequently, a comprehensive preoperative and perioperative assessment of the tumor is essential to determine its extent, particularly in cases involving dumbbell-shaped lesions. This case report describes our experience in managing a patient with a dumbbell-shaped vestibular schwannoma, where we achieved near-total resection while successfully preserving the patient’s facial nerve and hearing function. The early postoperative evaluation revealed no morbidity, and the patient experienced a significant improvement in their symptoms.

## Introduction

Vestibular schwannoma is a slow-growing tumor from the Schwann cell sheath along the vestibular nerve. This tumor accounts for nearly 80% of cerebellopontine angle (CPA) tumors in 0.7–1.0 people per 100 000 population [1]. Typically, Vestibular schwannoma arises from the intrameatal portion, expands into the CPA, and may involve other cranial nerves surrounding the vestibular nerve, then compress the brain stem. Vestibular schwannoma that expands inside the meatus and cerebellopontine region will have two globular segments and reveal a “dumbbell-shaped” lesion. This type of lesion poses difficulty in preserving the hearing function of the patients [2]. The main goals in treating vestibular schwannoma are total tumor removal with preservation of cranial nerve function, especially facial and vestibular nerve. Several approaches are used to achieve these goals by using retro sigmoid, middle fossa, or translabyrinthine with each own limitation and with the help of modern neurosurgical technology [3]. Hereby, we present a case of vestibular schwannoma in a 56-year-old woman that was removed using a retrosigmoid approach by mentioning its technical notes.

## Case report

A 56-year-old female patient came to our clinic complaining about dizziness. She has had a history of tinnitus in her left ear for 7 years. She also has had a hearing disturbance in the left ear for 3 years. On examination, she was fully alert with severe dizziness, and we found no facial nerve palsy with House-Brackmann Grade III with tinnitus on the left ear and loss of hearing sensation on the same side.

**Figure 1:**
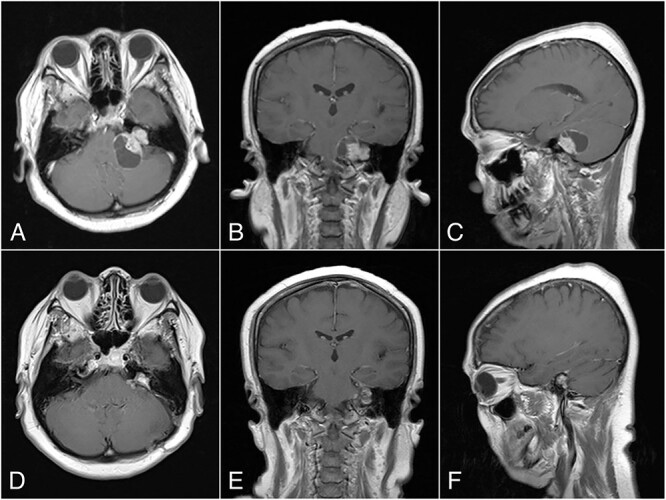
Preoperative MRI, (A) Axial, (B) Coronal, and (C) Sagittal plane, shows a “dumbbell” shaped vestibular schwannoma. Postoperative MRI, (D) Axial, (E) Coronal, and (F) Sagittal plane, shows near total resection with some residual mass on the left CPA.

Magnetic resonance imaging (MRI) with contrast revealed a large CPA tumor with two globular segments, one in the cerebellopontine region that compresses the brainstem and the fourth ventricle without hydrocephalus (Fig. 1A–C) and the other is inside the meatus that is suitable for a “dumbbell” vestibular schwannoma.

### Operative procedure

The patient lay in a park bench position toward the right side. The c-shaped incision was used to expose the retromastoid region. Asterion was identified, and the bone flap was opened. The transverse-sigmoid junction is identified, followed by a dural incision from the inferior part. Cerebrospinal fluids were drained from the left lateral medullary cistern before the dura completely opened to prevent cerebellar herniation from the dura opening. The tumor was identified and devascularized from its lateral portion.

**Figure 2:**
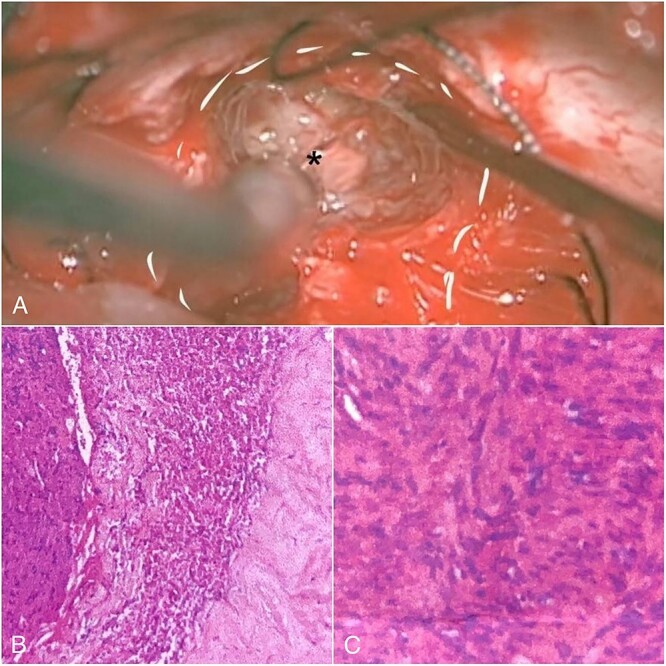
Intraoperative view and histopathological feature. (A) After debulking of the tumor, IAC was opened, and the intrameatal globular part (asterisk) could be appreciated and resected. (B) Histopathological findings show tumor cells with blood vessel proliferation that consist of collagen. (C) Schwann cells mixed with neuroma cells making a palisade-shaped structure.

We carefully identified the tumor border’s inferior pole and used a surgical cottonoid to mark the border between the lower cranial nerve. This technique will help us as a mark while performing tumor resection to prevent lower cranial nerve injury. The tumor removal process was performed piece by piece using micro scissors to better visualize the CPA region, thus minimizing brain retraction and safely dissecting the tumor from the brainstem. Anatomically, the facial nerve has already been severely disrupted by the tumor, and the capsule has adhered to the facial nerve with moderate preoperative facial nerve dysfunction. Then, we decided to leave some tumors surrounding the nerve fibers to prevent anatomical injury of the facial nerve. Meticulous dissection around the tumor using intraoperative monitoring (IOM) was performed to preserve the facial nerve. Drilling the internal auditory canal (IAC) using a high-speed drill is mandatory as removing the intrameatal part of the dumbbell schwannoma should start from normal nerve fibers. Resection was continued to detach the tumor from the lower cranial nerve and brainstem (Fig. 2A). Duraplasty was done, and the bone flap was placed back.

The histopathological finding shows the characteristics of a cystic vestibular schwannoma (Fig. 2B and C). Postoperative imaging evaluation using a computed tomography scan revealed no intracranial hemorrhage or hydrocephalus. On the sixth month of evaluation, the patient could do a regular activity without any significant complaints. Postoperative imaging evaluation showed a residual mass surrounding the facial nerve (Fig. 1D–F). After a discussion with the patient, we planned to observe this lesion as it did not cause any significant symptoms.

## Discussion

The main goals of the surgery in vestibular schwannoma are intraoperative anatomical preservation and postoperative functional preservation. The anatomical preservation of neural structures poses a challenge in vestibular schwannoma surgery. This region has numerous neurovascular structures, specifically between the pons, cerebellum, and middle cerebellar peduncle [3, 4]. Facial nerve preservation may be further differentiated into anatomical or functional preservation. Koos *et al*. found in their study that vestibular schwannoma with neurotopographic grade 1 tended to have the best facial nerve function either functionally or anatomically and was easier to dissect during surgery. Type 2 vestibular schwannoma that separates the section of the vestibular is more challenging to dissect as the capsule is embedded in the nerve fibers [5]. The intrameatal part of the tumor should be exposed by drilling the petrous bone. In case the entrance of IAC is barely visible, it can be confirmed by using a blunt hook [6].

In this case, we successfully preserve the patient’s facial nerve and hearing function using a retrosigmoid lateral suboccipital approach. Several techniques may help us to achieve this goal, such as (i) continuous irrigation, (ii) minimalizing the usage of bipolar cautery and Cavitron Ultrasound Surgical Aspirator (CUSA), (iii) perineural sheath preservation, (iv) drilling the IAC, and (v) IOM usage. Continuous saline irrigation has several benefits. It will provide good visualization of the surgical field, reducing blood loss yet avoiding complications [7]. Minimalizing bipolar cauterization is essential to prevent thermal injury of the nerve. On histopathological examination in animal models, this effect showed that the neural structure was damaged. This effect may be minimized by continuous irrigation that provides a thermoprotective effect. Even though continuous irrigation did not show its capacity to decrease the temperature during cauterization, it rapidly reverses the temperature [8].

CUSA application is related to neural damage proportional to the duration and intensity. Several complications, such as nerve fiber disruption, mild hemorrhage, and cortical disruption, might occur even with moderate intensity. Thus, the application of CUSA around the nerve must be minimized. Finally, intraoperative neuromonitoring with facial nerve stimulation is essential to assist the surgeon in identifying the nerve fibers, especially in large and dumbbell-shaped tumors that encase the facial nerve significantly [3, 9].

## References

[1] Nayak PK, Kumar RVS. Retromastoid-sub occipital: a novel approach to cerebello pontine angle in acoustic neuroma surgery-our experience in 21 cases. J Neurosci Rural Pract 2011;2:23. 21716801 10.4103/0976-3147.80084 PMC3122980

[2] Salzman KL, Davidson HC, Harnsberger HR, Glastonbury CM, Wiggins RH, Ellul S. et al. Dumbbell Schwannomas of the internal Auditory Canal. AJNR Am J Neuroradiol 2001;22:1368. 11498429 PMC7975197

[3] Atlas of neurosurgical techniques, 2-Vol. set. Am J Neuroradiol 2008;29:e51–2.

[4] Matsushima K, Yagmurlu K, Kohno M, Rhoton AL. Anatomy and approaches along the cerebellar-brainstem fissures. J Neurosurg 2016;124:248–63. 26274986 10.3171/2015.2.JNS142707

[5] Propp JM, McCarthy BJ, Davis FG, Preston-Martin S. Descriptive epidemiology of vestibular schwannomas. Neuro Oncol 2006;8:1–11. 16443943 10.1215/S1522851704001097 PMC1871924

[6] Koos WT, Day JD, Matula C, Levy DI. Neurotopographic considerations in the microsurgical treatment of small acoustic neurinomas. J Neurosurg 1998;88:506–12. 9488305 10.3171/jns.1998.88.3.0506

[7] Nagarajah D, Kueh YC, Lazim NM, Abdullah B. The hemostatic effect of hot saline irrigation in endoscopic sinus surgery: a systematic review and meta-analysis. Syst Rev 2022;11:246(1-10). 36401259 10.1186/s13643-022-02113-0 PMC9675124

[8] Donzelli J, Leonetti JP, Wurster RD, Lee JM, Young MRI. Neuroprotection due to irrigation during bipolar cautery. Arch Otolaryngol - Head Neck Surg 2000;126:149–53. 10680864 10.1001/archotol.126.2.149

[9] Kulwin CG, Cohen-Gadol AA. Technical nuances of resection of giant (> 5 cm) vestibular schwannomas: pearls for success. Neurosurg Focus 2012;33:E15. 10.3171/2012.7.FOCUS12177 22937849

